# Whole Genome Sequence Analysis of the First Australian OXA-48-Producing Outbreak-Associated *Klebsiella pneumoniae* Isolates: The Resistome and *In Vivo* Evolution

**DOI:** 10.1371/journal.pone.0059920

**Published:** 2013-03-29

**Authors:** Björn A. Espedido, Jason A. Steen, Helen Ziochos, Sean M. Grimmond, Matthew A. Cooper, Iain B. Gosbell, Sebastiaan J. van Hal, Slade O. Jensen

**Affiliations:** 1 Antibiotic Resistance and Mobile Elements Group, School of Medicine, University of Western Sydney, New South Wales, Australia; 2 Ingham Institute for Applied Medical Research, New South Wales, Australia; 3 Queensland Centre for Medical Genomics, University of Queensland, Queensland, Australia; 4 Institute for Molecular Bioscience, University of Queensland, Queensland, Australia; 5 Sydney South Western Pathology Service, NSW Pathology, New South Wales, Australia; 6 Department of Microbiology and Infectious Diseases, Royal Prince Alfred Hospital, New South Wales, Australia; Charité, Campus Benjamin Franklin, Germany

## Abstract

Whole genome sequencing was used to characterize the resistome of intensive care unit (ICU) outbreak-associated carbapenem-resistant *K. pneumoniae* isolates. Importantly, and of particular concern, the carbapenem-hydrolyzing β-lactamase gene *bla*_OXA-48_ and the extended-spectrum β-lactamase gene *bla*_CTX-M-14_, were identified on a single broad host-range conjugative plasmid. This represents the first report of *bla*_OXA-48_ in Australia and highlights the importance of resistance gene surveillance, as such plasmids can silently spread amongst enterobacterial populations and have the potential to drastically limit treatment options. Furthermore, the *in vivo* evolution of these isolates was also examined after 18 months of intra-abdominal carriage in a patient that transited through the ICU during the outbreak period. Reflecting the clonality of *K. pneumoniae*, only 11 single nucleotide polymorphisms (SNPs) were accumulated during this time-period and many of these were associated with genes involved in tolerance/resistance to antibiotics, metals or organic solvents, and transcriptional regulation. Collectively, these SNPs are likely to be associated with changes in virulence (at least to some extent) that have refined the *in vivo* colonization capacity of the original outbreak isolate.

## Introduction

*Klebsiella pneumoniae* is a common cause of infections worldwide, both in community and hospital settings [1], [2]. Based on data from the Study for Monitoring Antimicrobial Resistance Trends (SMART), carbapenems remain the most effective treatment option for these infections, especially those caused by strains producing extended-spectrum β-lactamases (ESBLs) [1], [2]. While the occurrence of ESBL-producing *K. pneumoniae* infections has been variable over the past decade, there has been an overall increase in the number of these strains [1], [3]. The consequence of ESBL-associated infections is a greater reliance on carbapenems as one of the few remaining effective agents. Therefore, the emergence of carbapenem-resistant *K. pneumoniae* is particularly worrisome, as not only are treatment options limited but these infections are associated with increased morbidity and mortality [4], [5].

In Australia, carbapenem resistance in *K. pneumoniae* is uncommon and over the past decade has generally been secondary to the expression of metallo-β-lactamase (MBL) genes (specifically *bla*_IMP-4_) [6], in combination with changes in outer-membrane porins. Recently, two *K. pneumoniae* isolates have been reported that produce either the MBL NDM-1 [7] or an Ambler Class A KPC-type carbapenem-hydrolyzing β-lactamase [8]. Furthermore, with respect to *Enterobacteriaceae*, Ambler class D carbapenem-hydrolyzing β-lactamase (CHDL) genes have also recently emerged in Australia with the report of a clinical *K. pneumoniae* isolate carrying a plasmid with *bla*_OXA-181_ [9]. However, a related gene, *bla*_OXA-48_, which was first identified in a *K. pneumoniae* isolate from Turkey in 2001 [10], and that has spread to Africa, Asia and Europe, has not previously been detected in Australia [11]. The broad dissemination of *bla*_OXA-48_, which has largely been due to an association with plasmid-borne Tn*1999* or related transposons [11], is of major concern given the ease at which transmission and spread occurs and the subsequent consequence for therapy.

In this study we used whole genome sequencing to characterize the resistome of the first known OXA-48 producing carbapenem-resistant *K. pneumoniae* isolates following an introduction resulting in an outbreak in a metropolitan Sydney Intensive Care Unit (ICU). In addition, we examine the *in vivo* evolution of this strain based on recovery of the same isolate from an “outbreak” patient following 18 months of carriage.

## Methods

### Bacterial Strains, Growth Conditions and Antibiotic Resistance Profiles

Isolates: In 2010, a multi-drug carbapenem-resistant *K. pneumoniae* (Kp001) was introduced into the ICU of a Sydney Metropolitan Hospital by a patient recently returned from Egypt. Three additional patients acquired the organism over several months before termination of the outbreak. All four patients who developed an infection with this organism died. However, 18 months later, a similar *K. pneumoniae* isolate (Kp002) was recovered from the abdominal fluid of a patient (post-hernia repair) who had transited through the ICU at the time of the initial outbreak, despite negative rectal screening swabs at the time of the outbreak. Upon referral to a reference laboratory, both isolates were indistinguishable by either antibiotic resistance profiling or molecular diagnostics (pulsed-field gel electrophoresis and enterobacterial repetitive intergenic consensus sequence PCR; data not shown).

Bacterial strains used in this study are listed in Table 1. Bacterial strains were grown at 37°C in LB medium (Sigma-Aldrich; St. Louis, USA) or on plates containing LB medium and 1.5% w/v agar (Amresco; Solon, USA), unless otherwise stated. When required, media was supplemented with 100 µg mL^−1^ ampicillin (Amresco; Solon, USA) and/or 100 µg mL^−1^ rifampicin (Sigma-Aldrich; St. Louis, USA). Antibiotic resistance profiles were determined on a VITEK 2 AST-N149 card using the global and natural resistance interpretive criteria (bioMérieux; Marcy L’Étoile, FRA).

**Table 1:** Bacterial strains, plasmids and primers.

Strain, plasmid or primer	Genotype, relevant characteristics or sequence	Source
**Strains** *E. coli*		
Ec002	Rifampicin^R^ derivative of DH5α: F- *endA hsdR17 supE44 thi-1* λ*- recA1 gyrA96 relA1* φ80 *dLac*ZΔM15	This study
Ec003	Ec002 carrying pJEG011 and pJEG012	This study
*K. pneumoniae* Kp001	Clinical outbreak-associated isolate carrying pJEG011 and pJEG012	This study
Kp002	Clinical outbreak-associated isolate phenotypically and genotypically indistinguishable from Kp001: isolated after 18 months of intra-abdominal carriage	This study
**Plasmids**		
pJEG011	IncL/M, Tn*1999*, IS*Ecp1*-*bla*_CTX-M-14_, Tn*5393j*Δ	This study
pJEG012	*pir*, Tn*1331*	This study
**Primers**		
aacA4-F	5′- gaagagtatgagtattcaaacatttcc -3′	This study
aacA4-R	5′- ggaagggttaggcaacac -3′	This study
aacC2-F	5′- gtttagaggagatatcgcgatg-3′	This study
aacC2-R	5′- ttgtcgacggcctctaac-3′	This study
CTX-M-14-F	5′- gagagtgcaacggatgatg -3′	This study
CTX-M-14-R	5′- tgcggctgggtaaaatag -3′	This study
OXA-48-F	5′- gcttccaccctaatttgatg -3′	This study
OXA-48-R	5′- cgagcatcagcattttgtc -3′	This study

### DNA Manipulations

DNA was extracted from *K. pneumoniae* and *Escherichia coli* cells using the ISOLATE Genomic DNA and Plasmid Mini Kits (Bioline; London, UK), respectively. DNA fragments were PCR-amplified using BioTaq (Bioline; London, UK) and primer pairs described in Table 1. Capillary sequencing of PCR products was performed by Macrogen Inc (Seoul, KOR).

### Whole Genome Sequencing

Kp001 DNA was sent to The Ramaciotti Centre (University of New South Wales; Sydney, AUS) for sequencing on an Illumina HiSeq 2000 system (Illumina Inc; San Diego, USA). A fragment library of Kp002 DNA was generated and sequenced on an Ion Torrent PGM (Life Technologies; Carlsbad, USA) according to the manufacturer’s instructions. Analysis of Kp001 and Kp002 genomic data was performed using CLC Genomics Workbench 5.5 (CLC bio; Katrinebjerg, DEN). Reference mapping of reads to the genome of the non-multi-drug resistant *K. pneumoniae* strain NTUH-K2044 (GenBank accession no. AP006725) facilitated variant analysis using a quality-based algorithm (as implemented in CLC Genomics Workbench) applying an 80% genotype frequency cutoff with a minimum coverage of 10 reads. Homopolymer variants as well as variants present in both Kp001 and Kp002 were excluded. The remaining variants were curated manually to ensure accurate identification. Raw data for this project has been uploaded to the Sequencing Read Archive under accession number SRA062913. A *de novo* assembly of reads that did not map to NTUH-K2044 was used to query an in-house database (constructed within CLC Genomics Workbench) of clinically relevant antibiotic resistance genes (see Table S1). BLASTn analyses of resultant contigs using the NCBI non-redundant nucleotide database were also performed in order to examine the genetic context of identified resistance determinants. Gaps in plasmid read mappings were closed via PCR amplification and capillary sequencing.

### Conjugation Experiments

Filter-based conjugation experiments were performed as previously described [12] using a rifampicin-resistant mutant *Escherichia coli* DH5α strain (Ec002), which was obtained via growth on an LB agar plate containing 100 µg mL^−1^ rifampicin.

## Results and Discussion

### Defining the Common Resistome

#### The mobile resistome

Both Kp001 and Kp002 were shown to be resistant to aminoglycosides and most β-lactams including, and of particular concern, meropenem. In order to fully define the resistome for both isolates, WGS reads that did not map to NTUH-K2044 were *de novo* assembled and examined via BLASTn analysis using an in-house database (created within CLC Genomics Workbench 5.5) of known antibiotic resistance determinants in Gram-negative bacteria (see Methods). Included in the local resistance gene database (RGD) were representative alleles of common aminoglycoside resistance genes [13], [14], quinolone resistance genes [15] and β-lactam resistance genes, including those that encode extended-spectrum and carbapenem-hydrolyzing β-lactamases [16]–[20]. Further BLASTn analysis and reference mapping was then performed in order to determine the genetic context of the identified determinants.

**Figure 1:**
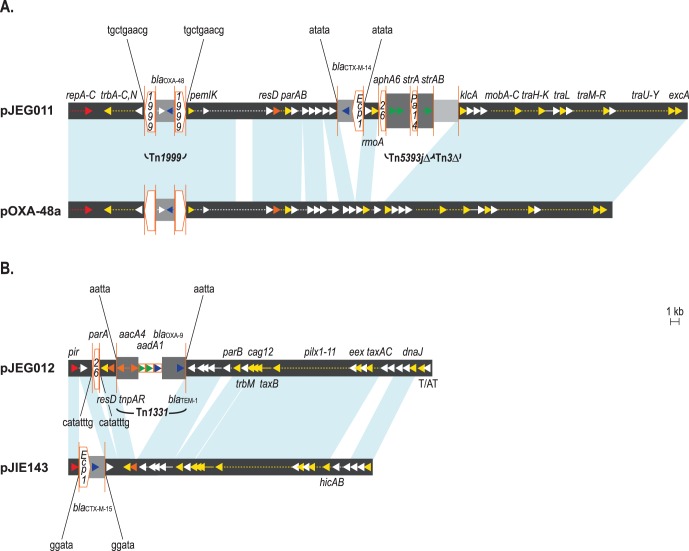
Structural features of plasmids pJEG011 and pJEG012. Comparisons to related plasmids pOXA-48a (A) and pJIE143 (B) are shown, respectively; plasmid backbones are represented by thick gray lines and areas of ≥99% sequence identity between plasmids are indicated by the light blue areas. Only the following selected genes are annotated and represented by colored arrows: plasmid replication genes, red; transposon-related genes, orange; plasmid partitioning, maintenance (*e.g.*, toxin/antitoxin systems (T/AT)), mobilization and conjugation genes, yellow; aminoglycoside resistance genes, green; β-lactam resistance genes, blue. Dashed arrows represent more than one gene or open reading frame. Insertion sequences (IS) are represented by orange pentagons with the IS number indicated within; the direction of the IS with respect to the transposase gene is indicated by the point of the pentagon. Inverted repeats associated with IS and transposons are indicated by vertical orange lines; the nucleotide sequences of the direct repeats resulting from IS and transposon insertion are indicated above or below the plasmid figures. Integron gene cassettes are represented by orange rectangles.

Based on interrogation of the RGD, four β-lactamase genes were identified in both Kp001 and Kp002: *bla*_SHV-1_ (which is ubiquitous in *K. pneumoniae*), *bla*_CTX-M-14_ (which differs from *bla*_CTX-M-9_ by 4 nucleotides), *bla*_OXA-9_ and, of particular importance, the *bla*_OXA-48_ CHDL gene. Further analysis revealed *bla*_CTX-M-14_ was present as part of an IS*Ecp1* transposition unit which had inserted into a plasmid designated pJEG011 (GenBank accession no. KC354801). pJEG011 shares >95% nucleotide sequence similarity with the backbone structure of the multi-resistance IncL/M conjugative plasmid pOXA-48a (GenBank accession no. JN626286) (11), including Tn*1999* containing the *bla*_OXA-48_ gene [21] (Figure 1A).

The aminoglycoside resistances genes *aphA6* and *strAB* were also present as part of pJEG011, and were located within a novel Tn*5393* module (Figure 1A). The other aminoglycoside resistance genes detected (*aacC2, aacA4* and *aadA1*) were not part of pJEG011. Further analysis revealed that the *aacC2* gene was located on a contig that is flanked by two copies of IS*26* in the same orientation as previously described for certain IncFII plasmids [22], however the genetic context of this determinant remained unclear. In contrast, both the *aacA4* and *aadA1* genes were located on the same contig along with *bla*_OXA-9_, and these were all present as part of Tn*1331*. Further examination of this contig revealed that Tn*1331* had inserted into a *pir*-type plasmid designated pJEG012 (GenBank accession no. KC354802), which shares >85% nucleotide sequence similarity with the backbone structure of another plasmid, pJIE143 (GenBank accession no. JN194214) [23]; pJEG012 also contained a putative toxin/anti-toxin system and a single copy of IS*26* (Figure 1B).

#### Contribution of single nucleotide variants

Both isolates had SNPs in *gyrA* and *parC*, which encode subunits of DNA gyrase and topoisomerase IV, respectively, and are involved in DNA replication and segregation. These SNPs result in amino acid changes within the quinolone resistance determining regions of GyrA (S80I) and ParC (S83Y and D87G), and collectively are known to confer high-level resistance to ciprofloxacin and nalidixic acid [24].

### Transfer of the Mobile Resistome

Conjugation experiments revealed that pJEG011 and pJEG012 could be readily transferred from Kp001 to Ec002. The presence of both plasmids in a single *E. coli* transconjugant, Ec003, was determined by PCR amplification of *bla*_CTX-M-14_, *bla*_OXA-48_ and *aacA4* (Table 2). The *aacC2* gene was not detected by PCR in the transconjugant (in agreement with the above analysis), suggesting that it is most likely located on the chromosome. Despite increased β-lactam MICs, Ec003 was fully susceptible to meropenem (Table 2). This was not unexpected as OXA-48 only hydrolyzes carbapenems at low levels [10].

**Table 2:** β-lactam MICs and PCR results for Kp001 and Ec003.

Antibiotic^a^	MICs (mg/L)^b^		
	Kp001	Ec003 (Ec002 transconjugant)	Ec002
Ampicillin	≥32	≥32	≤2
Amoxicillin/CLA	≥32	16	≤2
Ticarcillin/CLA	≥128	≥128	≤8
Piperacillin/TZB	≥128	≥128	≤4
Cefazolin	≥64	≥64	≤4
Cefoxitin	≥64	≤4	≤4
Ceftazidime	4	≤1	≤1
Ceftriaxone	≥64	32	≤1
Cefepime	≥64	≤1	≤1
Meropenem	≥16	≤0.25	≤0.25
Gene Target	PCR Results		
*aacA4*	+	+	−
*bla*_CTX-M-14_	+	+	−
*bla*_OXA-48_	+	+	−
*aacC2*	+	−	−

a Antibiotic abbreviations: CLA, clavulanic acid, TZB, tazobactam.

b MICs were determined using a VITEK 2 AST-N149 card.

In *K. pneumoniae*, carbapenem resistance in the setting of OXA-48 production is usually co-dependent upon the presence of additional mechanisms of resistance, such as outer membrane porin defects. These mutations generally occur within the OmpK35 and OmpK36 porins, which allow carbapenem entry into the cell. Analysis of the isolates revealed that *ompK35* was truncated via a 485 bp chromosomal deletion (nt 1,880,269–1,880,753) while *ompK36* contained a duplication of the sequence GGCGAC (nt 1,879,495–1,879,500). This duplication would most likely result in partial occlusion of the OmpK36 channel as a result of insertion of two additional amino acids into loop 3 [25].

### *In vivo* Evolution

Kp001 and Kp002 were considered identical based on their antibiotic resistance profiles (Table 3), molecular (see methods; data not shown) and *in silico* multi-locus sequence typing [26] which revealed that both isolates belonged to ST101. Nucleotide variant analysis revealed that Kp001 and Kp002 differed by 11 single nucleotide polymorphisms (SNPs; Table 4), many of which are associated with proteins involved in tolerance/resistance to antibiotics, metals or organic solvents, and transcriptional regulation.

**Table 3:** Antibiotic MICs for Kp001 and Kp002.

Antibiotic^a^	MICs (mg/L)^b^		
	Kp001	Kp002	
Ampicillin	≥32	≥32	
Amoxicillin/CLA	≥32	≥32	
Ticarcillin/CLA	≥128	≥128	
Piperacillin/TZB	≥128	≥128	
Cefazolin	≥64	≥64	
Cefoxitin	≥64	≥64	
Ceftazidime	4	4	
Ceftriaxone	≥64	≥64	
Cefepime	≥64	≥64	
Meropenem	≥16	≥16	
Amikacin	≥64	≥64	
Gentamicin	≥16	≥16	
Tobramycin	≥16	≥16	
Nalidixic acid	≥32	≥32	
Ciprofloxacin	≥4	≥4	
Norfloxacin	≥16	≥16	
Trimethoprim	1	1	
TMP/SXT	≤20	≤20	

a Antibiotic abbreviations: CLA, clavulanic acid, TZB, tazobactam; TMP/SXT, trimethoprim/sulfamethoxazole.

b MICs were determined using a VITEK 2 AST-N149 card.

**Table 4:** SNPs present in Kp002.

Mutation^a^	Gene or Locus^b^	Function	Amino Acid Change
C→A (98,127)	KP1_0101	putative LysR-type transcriptional regulator	T131N
A→G (228,794)	*rpoB*	RNA polymerase, β subunit	D527G
A→T (678,116)	*cusS*	copper-sensing two-component system sensor kinase	S446C
C→T (1,767,550)	*yliC*	ABC transport system periplasmic binding component	P256S
A→C (2,910,808)	*slyA*	Transcriptional regulator	V120G
C→T (2,960,076)	Upstream of KP1_3109	putative LysR-type transcriptional regulator	–
G→A (3,490,471)	*gyrI*	DNA gyrase inhibitor	A99V
C→A (3,721,779)	*glpC*	sn-glycerol-3-phosphate dehydrogenase K, small subunit	P383T
A→G (4,360,381)	Intergenic		–
G→A (4,380,149)	Intergenic		–
G→A (5,084,490)	KP1_5543	putative acetyltransferase	G120Stop

a Nucleotide change (genetic location in *K. pneumoniae* strain NTUH-K2044).

b Locus name as annotated in *K. pneumoniae* strain NTUH-K2044.

Compared to Kp001, a SNP in Kp002 was observed in a region of *rpoB* known to contribute to rifampicin resistance [27]. Subsequently, rifampicin resistance was demonstrated *in vitro* for Kp002, but not Kp001 (wild-type *rpoB*), as it could be cultured on LB agar containing 100 µg mL^−1^ rifampicin. In Australia, it is common practice to soak the surgical mesh in a solution of rifampicin prior to surgery as an infection prevention measure. This exposure most likely contributed to the *in vivo* selection of the *rpoB* mutation as Kp002 was isolated from the patients’ intra-abdominal mesh associated collection post hernia repair.

In Kp002, a SNP in *gyrI* resulted in an amino acid change that may affect protein activity and play a role in decreased quinolone susceptibility. Although overexpression of *gyrI* (aka *sbmC*), has been shown to confer protection against quinolones and toxin/anti-toxin plasmid maintenance systems in *E. coli* [28], it is unlikely to have had much additional effect in our isolate given the high level quinolone resistance mutations already present.

In the context of regulation, two SNPs were associated with LysR-type transcriptional regulators (LTTRs), which represent the largest group of transcriptional regulators regulating genes/pathways associated with metabolism, motility, quorum sensing and virulence [29]. The amino acid change in the gene product of locus KP1_0101 is flanked by amino acids involved in dimerization, based on the conserved domain database [30], suggesting that this mutation is likely to have functional significance. Furthermore, the mutation upstream of the other putative LTTR gene (locus KP1_3109; Table 4) may have a bearing on promoter activity, as it is located 35 bp upstream of the start codon. In addition, there is also a SNP present in *slyA*, which encodes a known transcriptional regulator of virulence genes. SlyA is involved in conferring resistance to antimicrobial peptides and oxidative stress in salmonellae [31], [32] as well as regulation of fimbriae in *E. coli*, which have an important role in colonization and pathogenesis [33]; based on the crystal structure of SlyA, the resulting amino acid change (V120G) is located between two α-helices involved in dimerization [34]. Although the functional consequences of these mutations are not directly known, it is interesting that they occurred during 18 months of intra-abdominal carriage after an initial outbreak event that resulted in patient deaths. As such, it is likely that they are collectively associated with changes in virulence (at least to some extent) that have refined the *in vivo* colonization capacity of Kp002. In this context it is relevant to note that Young *et al.*, [35] recently suggested that truncation of a *Staphylococcus aureus* transcriptional regulator (implicated in pathogenicity) after 13 months of carriage, was a key factor driving changes in virulence capacity.

### Concluding Remarks

To the best of our knowledge, this study represents the first report of the *bla*_OXA-48_ CHDL gene in Australia. This study also illustrates the *in vivo* evolution of a multidrug-resistant *K. pneumoniae* isolate during 18 months of carriage. Of note, some of the SNPs identified, particularly those associated with transcriptional regulators, may be involved in modulation of Kp002 virulence capacity. In a global context, this is also the first report of *bla*_CTX-M-14_ and *bla*_OXA-48_ co-residing on a single broad host-range conjugative plasmid (*i.e.*, pJEG011). While international travel has facilitated the clonal spread of *bla*_OXA-48_-containing *K. pneumoniae* ST101 isolates [36]–[38], especially from countries along the Mediterranean Sea, the presence of *bla*_OXA-48_ within Tn*1999* (and related transposons) on different Inc group plasmids [21], [39], [40], has played a crucial role in its dissemination. The emergence of plasmids such as pJEG011, and the one recently described by Potron *et al.* [41], is of great clinical concern as they have the potential to more broadly disseminate resistance associated with these determinants. In this respect, it is also concerning that these determinants have the potential to go undetected based on antibiotic susceptibility profiles. Therefore, this study highlights the importance of surveillance based on resistance screening, especially in environments where antibiotic selection pressure is prevalent.

## Supporting Information

Table S1 **List of antibiotic resistance genes used in the in-house database for resistome determination.**
(XLSX)
